# Seroprevalence of IgG antibodies against SARS-CoV-2 – a serial prospective cross-sectional nationwide study of residual samples, Belgium, March to October 2020

**DOI:** 10.2807/1560-7917.ES.2022.27.9.2100419

**Published:** 2022-03-03

**Authors:** Sereina Annik Herzog, Jessie De Bie, Steven Abrams, Ine Wouters, Esra Ekinci, Lisbeth Patteet, Astrid Coppens, Sandy De Spiegeleer, Philippe Beutels, Pierre Van Damme, Niel Hens, Heidi Theeten

**Affiliations:** 1 Centre for Health Economics Research and Modelling of Infectious Diseases (CHERMID), Vaccine & Infectious Disease Institute (VAXINFECTIO), University of Antwerp, Wilrijk, Belgium; 2 Institute for Medical Informatics, Statistics and Documentation, Medical University of Graz, Graz, Austria; 3 Centre for the Evaluation of Vaccination, Vaccine and Infectious Disease Institute, University of Antwerp, Wilrijk, Belgium; 4 Global Health Institute (GHI), Family Medicine and Population Health (FAMPOP), University of Antwerp, Wilrijk, Belgium; 5 Data Science Institute (DSI), Interuniversity Institute for Biostatistics and statistical Bioinformatics (I-BioStat), UHasselt, Hasselt, Belgium; 6 Algemeen Medisch Laboratorium (AML), Sonic Healthcare, Antwerp, Belgium; 7 Laboratoire Luc OLIVIER, Fernelmont, Belgium

**Keywords:** serial sero-survey, COVID-19, seroprevalence seroincidence, ELISA, SARS-CoV-2 S1 epitope, humoral immunity

## Abstract

**Background:**

To control epidemic waves, it is important to know the susceptibility to SARS-CoV-2 and its evolution over time in relation to the control measures taken.

**Aim:**

To assess the evolving SARS-CoV-2 seroprevalence and seroincidence related to the first national lockdown in Belgium, we performed a nationwide seroprevalence study, stratified by age, sex and region using 3,000–4,000 residual samples during seven periods between 30 March and 17 October 2020.

**Methods:**

We analysed residual sera from ambulatory patients for IgG antibodies against the SARS-CoV-2 S1 protein with a semiquantitative commercial ELISA. Weighted seroprevalence (overall and by age category and sex) and seroincidence during seven consecutive periods were estimated for the Belgian population while accommodating test-specific sensitivity and specificity.

**Results:**

The weighted overall seroprevalence initially increased from 1.8% (95% credible interval (CrI): 1.0–2.6) to 5.3% (95% CrI: 4.2–6.4), implying a seroincidence of 3.4% (95% CrI: 2.4–4.6) between the first and second collection period over a period of 3 weeks during lockdown (start lockdown mid-March 2020). Thereafter, seroprevalence stabilised, however, significant decreases were observed when comparing the third with the fifth, sixth and seventh period, resulting in negative seroincidence estimates after lockdown was lifted. We estimated for the last collection period mid-October 2020 a weighted overall seroprevalence of 4.2% (95% CrI: 3.1–5.2).

**Conclusion:**

During lockdown, an initially small but increasing fraction of the Belgian population showed serologically detectable signs of exposure to SARS-CoV-2, which did not further increase when confinement measures eased and full lockdown was lifted.

## Introduction

By August 2021, globally over 213.8 million people were confirmed to be infected with severe acute respiratory syndrome coronavirus 2 (SARS-CoV-2) causing coronavirus disease (COVID-19) [1]. Clinical symptoms caused by the infection include loss of taste and smell, fever, malaise, dry cough, shortness of breath and respiratory distress. Reported illnesses have ranged from very mild to severe (from progressive respiratory failure to death) [2]. In addition, increasing age, male sex, smoking and comorbidities such as cardiovascular diseases and diabetes have been identified as risk factors for developing severe illness [3].

Until mid-October 2020, the date of the last collection period in this study, there was no vaccine or effective cure available to protect against or treat COVID-19. Therefore, many countries considered unprecedented measures such as physical distancing, large-scale isolation and closure of borders, schools and workplaces in order to mitigate the spread of the disease and reduce the pressure on the healthcare system.

In Belgium, the first confirmed COVID-19 case was reported on 4 February 2020, an asymptomatic person repatriated from Wuhan, China [4]. The first locally transmitted cases were confirmed on 2 March 2020. Thereafter, the number of confirmed COVID-19 cases increased rapidly. Belgium is a densely populated European country with, in 2020, ca 11.49 million inhabitants (374 persons/km^2^). It consists of three regions: Flanders (487 inhabitants/km^2^), Wallonia (216 inhabitants/km^2^) and the Brussels Capital Region (7,501 persons/km^2^) [5]. The Belgian Scientific Institute for Public Health, Sciensano, reported that by 17 October 2020, 242,217 COVID-19 cases were confirmed (2.1% of the Belgian population; 5.8% of the tested individuals) and 10,410 had died. The most affected age group regarding reported cases was 20–29 years (18.3% of the age group; 44,411/242,217) [6]. 

Importantly, the PCR testing capacity to diagnose SARS-CoV-2-infected people in Belgium was very limited during the first weeks of lockdown (2,000–3,000 tests/day) and gradually increased to a more adequate capacity in autumn 2020 (10,000–20,000 tests/day) [6]. Therefore, more knowledge on and estimation of the age-specific susceptibility to SARS-CoV-2 and its evolution over time, related to control measures that have been taken, is important to guide policymakers aiming to control the current and potential future epidemic waves of COVID-19. These needs were translated into the following research objectives: (i) to establish a national serum bank with residual samples on a periodic basis (cross-sectional study design) in order to estimate the seroprevalence and seroincidence in Belgium and to follow up trends herein over time and (ii) to estimate the age-specific prevalence of SARS-CoV-2 antibodies.

## Methods

### Study design

**Figure 1:**
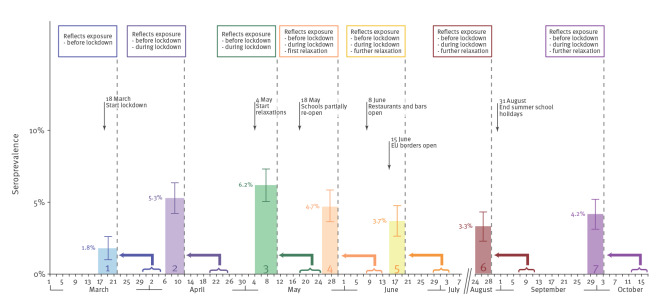
Collection periods related to COVID-19 confinement measures taken in Belgium, 2020

COVID-19: coronavirus disease; EU: European Union.

This prospective cross-sectional nationwide seroprevalence study using residual samples was conducted in individuals aged 0–101 years. In each collection period, sera were collected over a period of 1 week. The seven collection periods represent different exposure periods: (i) 30 March to 5 April 2020 mainly reflects exposure before the lockdown; (ii) 20 to 26 April 2020 and (iii) 18 to 25 May 2020 additionally reflect exposure during full lockdown; (iv) 8 to 13 June 2020 additionally reflects exposure during the period of first relaxation of confinement measures (partial re-opening of schools); (v) 29 June to 4 July 2020 additionally reflects changes during further relaxations (re-opening of shops, restaurants and bars); (vi) 7 to 12 September 2020 and (vii) 12 to 17 October 2020 are collection periods after the Belgium summer school holidays (see also Figure 1).

Collection periods 1 to 7 are indicated with curly brackets whereas the weighted overall seroprevalence estimates are displayed 14 days earlier in order to reflect the minimum time needed to build up IgG antibodies against SARS-CoV-2 that can be detected by ELISA tests. The bar height shows the weighted overall seroprevalence estimate and the interval the corresponding 95% credible interval.

A serum bank covering all Belgian regions was established by collecting residual sera from 10 private diagnostic laboratories. The majority of residual samples originated from two large routine laboratories in Flanders (AML) and Wallonia (laboratoire Luc OLIVIER), each with a large geographical network covering the whole of Belgium. These two laboratories have a high daily throughput of blood samples and received each up to 23,000 samples per week during the study period for a variety of diagnostic tests. Each laboratory was allocated a fixed number of samples per age group (10-year age bands, oldest age group ≥ 90 years) per region (Wallonia, Flanders, Brussels) and per collection period. The number of samples was stratified by sex within each age group.

Residual serum samples in this study originated from ambulatory patients (including people living in nursing homes) visiting their doctor (mainly general practitioners) for any reason including primary care, routine check-up or follow-up of pathology. The samples needed to have a minimum volume of 0.5 mL (no haemolysis requirements). To avoid disproportionate selection of subjects with acute and/or severe illness including COVID-19, samples originating from hospitals and triage centres were excluded from the study. Further background information on the residual samples was not available, except for COVID-19 diagnostics. We calculated the percentage of COVID-19 diagnostic tests performed within 2 weeks before collection of the residual samples in this study in order to check for over-representation of residual samples from patients who thought they were infected with COVID-19. Only 1–2% of the residual samples collected in periods 1 and 2 (PCR test) and 6–8% of the residual samples in periods 3–7 (PCR and/or serology test) were linked to COVID-19 diagnostic testing.

### Sample size

We calculated the sample size per periodical collection according to: (i) previous experience with various age-specific analyses of seroprevalence data in Belgium [7], (ii) estimates of the number of SARS-CoV-2-infected people in Belgium and (iii) the estimated evolution of the epidemic curve. Based on case numbers (hospitalised cases confirmed with COVID-19), we estimated the overall prevalence of SARS-CoV-2 infection at the start of the study at ca 0.4%. Therefore, a total sample size of 4,000 in the first collection period ensured the estimation of the overall prevalence with a margin of error of 0.2%; the precision regarding the age-specific prevalence estimates was lower because of the division of samples across the age groups. We expected an increase in prevalence during the study period and therefore planned 3,000 samples from the second collection period onwards. From collection period 2 onwards, we adapted target numbers per age group according to feasibility and sample availability and aimed at maximising precision and assessing the impact of a change in epidemic control policy.

### Sample preparation and analysis

The mean time between collection and centrifugation of blood samples was 4 h at room temperature. After centrifugation of blood samples, selected residual sera (minimum 0.5 mL) were routinely kept refrigerated (4–8 °C) and were selected for inclusion and testing within 14 days after collection, according to the manufacturer’s instructions (EuroImmun, Luebeck, Germany), and finally stored at −20 °C. The test kit used to obtain serology results was a semiquantitative test (EuroImmun), measuring IgG antibodies against the SARS-CoV-2 S1 protein in serum (ELISA). The test was performed as previously described by Lassaunière et al. [8]. A case–control validation study with 326 pre-pandemic negative controls and 181 RT-PCR-confirmed COVID-19 cases estimated 85.1% sensitivity and 98.8% specificity using the manufacturer’s recommended cut-off for positivity (optical density value ≥ 1.1) [9]. Presence of detectable IgG antibodies indicates prior exposure to SARS-CoV-2, an infection which may be resolved or is still resolving, and possibly protection against reinfection [8,10].

### Data management

Data collected for each sample included: unique sample code, sample date, age, sex and postal code of the place of residence. Samples were delivered anonymously to the investigators. Triage and check for duplicates was done per collection period in the collecting laboratories before anonymisation.

Serological results were linked to the database based on the sample code. No further data entry was required. All files were kept on a secured server at the University of Antwerp. Data will be stored for 20 years.

### Statistical analysis

The serostatus of an individual was considered to be positive if the measured IgG optical density values were ≥ 1.1, equivocal IgG values were considered negative following the manufacturer’s recommendations which were developed for clinical use. Descriptive analysis included mapping of sample origin as well as serostatus (crude figures) up to municipality level per collection period.

For all analyses, the overall seroprevalence, age-specific seroprevalence by 10-year age bands and seroprevalence by sex for each collection period were obtained as the posterior medians (with 95% credible interval (CrI)) of the corresponding posterior distributions for the probabilities to be seropositive. More specifically, we considered a Bayesian approach based on the immunological status (i.e. serostatus) of each individual following a Bernoulli distribution, thereby including individual-specific design weights. Moreover, the model accommodated test-specific sensitivity and specificity of the ELISA assay and uncertainty about those when estimating the seroprevalence. In order to inform these quantities, we relied on estimates obtained from the validation study described above [9]. The seroincidence estimates were obtained as the posterior medians (with 95% CrI) of the corresponding posterior distributions for the difference between the probabilities to be seropositive between collection periods. We performed analytical sensitivity analyses restricting the validation dataset to outpatients only and assuming perfect sensitivity and specificity. The model was implemented in Stan using the interface R (rstan version-2.21.1) [11-13]. We ran 6,000 iterations and assessed convergence visually and using the R-hat statistic (for more details on the statistical methodology, see the Supplement, section S1).

We assigned for each collection period weights to the samples such that they mimicked the Belgian population structure according to age, sex and provinces for 2020 [5]. Weights were computed by comparing the sample and population frequencies, i.e. we used a complete cross-frequency table for sex and 10-year age bands and a marginal distribution for the provinces. Weights were trimmed to a maximum value of 3 to reduce the influence of samples in under-represented strata (see Supplementary Figure S1 for details on the weighting). All analyses were done with the statistical software R (version 4.0.3; R Foundation, Vienna, Austria); to compute weights, we used the package survey (version 4.0) [14].

### Ethical statement

The study protocol was approved by the Ethics Committee of the University Hospital Antwerp-University of Antwerp on 30 March 2020 (ref 20/13/158; Belgian Number B3002020000047) and agreed with inclusion without informed consent, on the condition of the samples being collected unlinked and anonymously (see the Supplement for the study protocol).

## Results

**Figure 2:**
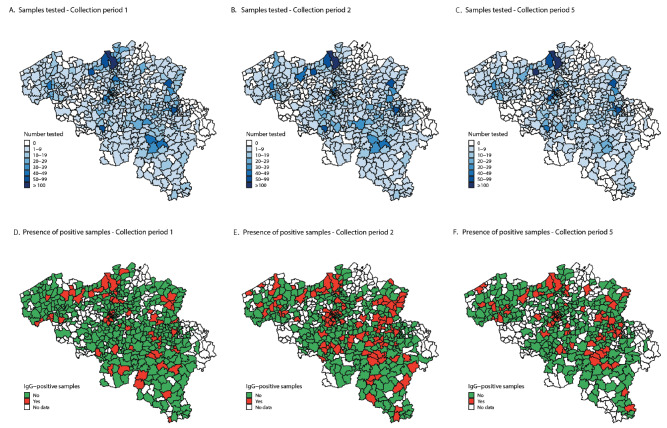
COVID-19 seroprevalence at municipality level for collection period 1, 2, and 5, Belgium, 2020 (n = 3,910; 3,397 and 3,023)

A total of 22,545 serum samples were collected over seven 1-week periods between 30 March and 17 October 2020 to measure the anti-SARS-CoV-2 IgG serostatus. The regional, age and sex distribution of these samples is shown in the Table (further details such as age distribution by sex are provided in Supplementary Figure S2); deviations from the population distribution were taken into account in the estimation of the weighted seroprevalences. Figure 2 shows in exemplary results for the collection periods 1, 2 and 5 that the origin of the samples was evenly distributed throughout Belgium (panels A–C) and that positive samples were spread over municipalities across Belgium (panels D–F). The complete results for all seven collection periods are provided in Supplementary Figures S3–S4.

**Table:** Study population, COVID-19 seroprevalence study, collection periods 1 to 7, Belgium, 2020 (n = 22,545)

		Collection period 1		Collection period 2		Collection period 3		Collection period 4		Collection period 5		Collection period 6		Collection period 7	
		30 Mar–5 Apr		20–26 Apr		18–25 May		8–13 June		29 June–4 July		7–12 Sept		12–17 Oct	
		n	%	n	%	n	%	n	%	n	%	n	%	n	%
Total number of samples		3,910		3,397		3,242		2,960		3,023		3,047		2,966	
Region	Wallonia	1,511	38.6	1,539	45.3	1,292	39.9	1,100	37.2	1,068	35.3	1,259	41.3	1,144	38.6
	Flanders	2,195	56.1	1,556	45.8	1,542	47.6	1,526	51.6	1,621	53.6	1,491	48.9	1,534	51.7
	Brussels	204	5.2	302	8.9	408	12.6	334	11.3	334	11.0	297	9.7	288	9.7
Age in years	< 10	36	0.9	85	2.5	174	5.4	124	4.2	110	3.6	68	2.2	68	2.3
	10–19	294	7.5	442	13.0	431	13.3	375	12.7	413	13.7	432	14.2	405	13.7
	20–29	436	11.2	375	11.0	414	12.8	383	12.9	394	13.0	406	13.3	402	13.6
	30–39	461	11.8	407	12.0	424	13.1	395	13.3	396	13.1	396	13.0	397	13.4
	40–49	468	12.0	406	12.0	411	12.7	394	13.3	403	13.3	399	13.1	397	13.4
	50–59	498	12.7	430	12.7	419	12.9	393	13.3	400	13.2	402	13.2	400	13.5
	60–69	507	13.0	426	12.5	417	12.9	399	13.5	403	13.3	403	13.2	406	13.7
	70–79	506	12.9	316	9.3	236	7.3	201	6.8	204	6.7	212	7.0	204	6.9
	80–89	493	12.6	315	9.3	163	5.0	166	5.6	160	5.3	167	5.5	160	5.4
	≥ 90	211	5.4	195	5.7	153	4.7	130	4.4	140	4.6	162	5.3	127	4.3
Sex	Male	1,799	46.0	1,599	47.1	1,587	49.0	1,425	48.1	1,471	48.7	1,500	49.2	1,377	46.4
	Female	2,111	54.0	1,798	52.9	1,655	52.0	1,535	51.9	1,552	51.3	1,547	50.8	1,589	53.6

COVID-19: coronavirus disease.

COVID-19: coronavirus disease.

Panels A–C: number of samples tested in each municipality; panels D–F: presence of IgG-positive vs exclusively IgG-negative samples in each municipality.

**Figure 3:**
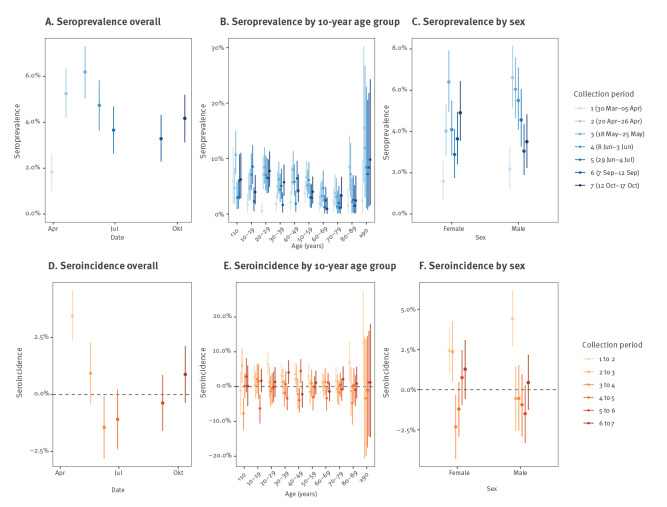
Weighted COVID-19 seroprevalence (A, B, C) and seroincidence (D, E, F) estimates in Belgium, 2020 (n = 22,545)

At the start, the seroprevalence estimates per age category ranged between 0.6% (20–30 years) and 5.9% (0–10 years) in collection period 1. The weighted overall seroprevalence showed a significant increase between collection period 1 and 2, i.e. from 1.8% (95% CrI: 1.0–2.6) to 5.3% (95% CrI: 4.2–6.4) over a period of 3 weeks (Figure 3, panel A) which is also shown by the overall seroincidence estimate of 3.4% (95% CrI: 2.4–4.6) (Figure 3, panel D). This significant increase in seroprevalence was reflected in the age categories 20–49, 80–89 and ≥ 90 as indicated by the seroincidence estimates (Figure 3, panels B and  E) and within each sex (Figure 3, panels C and F). The estimations displayed in Figure 3 can be found in Supplementary Tables S1 and S2.

COVID-19: coronavirus disease.

Points correspond to the estimates, lines to the 95% credible intervals. The seroincidence estimates indicate the difference in seroprevalence estimates between two consecutive collection rounds.

Compared with period 2, the overall seroprevalence did not increase thereafter as shown in Figure 3 (panel A). For the last collection period in mid-October, we estimated an overall seroprevalence of 4.2% (95% CrI: 3.1–5.2). However, we observed significant decreases when comparing the third period with the fifth, sixth and seventh, with seroincidences of −2.5% (95% CrI: −1.2 to −3.9), −2.9% (95% CrI: −1.6 to −4.2) and −2.0% (95% CrI: −0.7 to −3.4), respectively. For the first two comparisons, these decreases also occurred in three subgroups: age categories < 10 and 40–49 years, and in female subjects. Comparing male and female individuals, we observed a significantly higher seroprevalence estimate in period 2 for males (+2.6%; 95% CrI: 0.7–4.5), and from the second to the third period a higher seroincidence estimate for females (+2.9%; 95% CrI: 1.1–4.5). For these analyses, we estimated the diagnostic test-specific sensitivity and specificity at 84.9% (95% CrI: 82.9–86.9) and 98.7% (95% CrI: 98.2–99.1), respectively [9]. The results from the analytical sensitivity analysis, which investigated the influence of sensitivity and specificity, i.e. once restricting the validation dataset to outpatients only and once assuming perfect sensitivity and specificity, did not change the interpretation (Supplementary Figures S5-S10).

## Discussion

This study estimated seroprevalence and seroincidence of IgG antibodies against the SARS-CoV-2 S1 protein in Belgium based on residual sera collected in seven rounds from 30 March to 17 October 2020. The results give an indication of the state of the COVID-19 epidemic in Belgium, showing that only an estimated 1.8% (95% CrI: 1.0–2.6) of the population had detectable antibodies against SARS-CoV-2 at the start of lockdown (March 2020), which had more than doubled 3 weeks later (seroincidence: +3.4%; 95% CrI: 2.4–4.6). However, seroprevalence stabilised thereafter and decreased until the start of summer holidays (July), which was also reflected in negative seroincidence estimates. These seroprevalences continued in the same range, even after re-opening of the schools after summer resulting in a seroprevalence of 4.2% (95% CrI: 3.1–5.2) by mid-October.

Stringent containment measures were enforced in Belgium starting from 13 March 2020. These included travel bans and closure of borders, schools, shops, factories and social gatherings in an effort to contain the spread of COVID-19 and decrease the pressure on the healthcare system. These intervention measures slowed down the number of COVID-19 patients that were hospitalised daily. In the first 2 weeks of the lockdown (up to 25 March 2020), more than 500 cases were hospitalised daily, and this growth rate halved 4 weeks later [6]. By 26 April 2020, 0.1% of the Belgian population had been hospitalised for COVID-19 (14,822/11.46 × 10^6^) and 0.4% of the Belgian population had tested positive for SARS-CoV-2 (48,093/11.46 × 10^6^) in a total of 32,1862 screened patients [6]. The estimated seroprevalence (5.3%, 95% CrI: 4.2–6.4) in the same period (20–26 April 2020) indicates that far more people had generated antibodies against SARS-CoV-2 and thus had been in contact with the virus than what was expected from the number of confirmed COVID-19 cases reported in Belgium at that time. 

These seroprevalences provide insights into the dark number of SARS-CoV-2 infections, which is indeed a multiple of the confirmed cases. By end of June, the number of daily hospital admissions in Belgium dropped below 20 and the number of confirmed COVID-19 cases stabilised at a lower level than the estimated seroprevalence in Belgium but hospital admissions increased again by October. Clearly, the reported numbers of confirmed COVID-19 cases represent an underestimation and were influenced by the testing policy as testing was initially focused on the most severe symptomatic cases presenting to hospitals (see [6] and the detailed collection of numbers in Supplementary Table S3). Vice versa, also the seroprevalences in this study are possibly underestimated because residual samples from hospitals were excluded. Moreover, patients with upper respiratory tract infections were not allowed to visit general practitioners and ambulatory care during the lockdown period, possibly contributing to a further underestimation of the seroprevalences in collection periods 2, 3 and 4. The risk population, who possibly adhered better to self-confinement, as well as patients with non-urgent health problems were less likely to visit their doctor until later stages of the epidemic (collection periods 5–7). This may have resulted in a higher proportion of subjects not infected with SARS-CoV-2 for whom residual samples were analysed in the later collection periods, hence contributing, at least partly, to a significant drop in seroprevalences and seroincidence towards the fifth collection period (see also [15]). Regardless of this change in care seeking behaviour throughout the study, the current seroprevalence study in combination with the reported confirmed COVID-19 cases may form a useful tool to estimate the total number of recently acquired SARS-CoV-2 infections in Belgium. Moreover, this study gives an indication of the seroprevalence and seroincidence in light of the confinement measures taken in Belgium which helps understand the spread of SARS-CoV-2 and the significance of periodical variations.

From the above it is clear that determining of the extent of spread of SARS-CoV-2 at country level is a challenge. Moreover, the sensitivity of the serological test used in this study depends on the time since the onset of symptoms [8,16], thereby preventing a fraction of the infected subjects from testing seropositive if not infected long enough or too long before testing. By day 14 after symptom onset, IgG against SARS-CoV-2 are detectable in serum of the majority of patients [2]. Possibly, people recently infected with SARS-CoV-2, in whom antibodies were not yet detectable in blood, may have been included in the current study. Individuals with asymptomatic and pauci-symptomatic SARS-CoV-2 infections, who reportedly may develop low levels or no antibodies against SARS-CoV-2, may have been included in this study as well [17]. Some studies have reported that the decay of IgG levels starts within 2–3 months after infection [18-20] whereas others have reported long-lasting antibody response [21,22]. If IgG levels decrease within a few months, this could result in the situation that pauci-symptomatic people and those who had an infection more than a few months before testing may have received a seronegative test result and thus cause underestimation of the incidence of infection. Moreover, this would also imply that seroprevalence studies on SARS-CoV-2 would only be able to give information on the past few months.

A seroprevalence study conducted in healthy adult blood donors in Belgium has described, similar to this study, a doubling of seroprevalence estimates between end of March and mid-April 2020, which was followed by stable estimates around 5% by mid-September [23]. In Switzerland, a population-based household study conducted in Geneva also estimated a doubling of seroprevalence estimates within 5 weeks (6 April–9 May 2020) from 4.8% (95% confidence interval (CI): 2.4–8.0; n = 341) to 10·8% (95% CI: 8.2–13.9; n = 775) [15]. In Spain, a national seroprevalence of 5% (95% CI: 4.7–5.4; n = 61,075) was reported in the period from 27 April to 11 May 2020, comparable in magnitude with our study [24]. In the United Kingdom, the plateauing of seroprevalences has also been observed in a study with blood donors in June 2020 [25]. However, comparing seroprevalence estimates across countries is hampered for several reasons, for example differences in policy and intervention measures taken initially, the extent to which social activity levels and the social contact behaviour in general have changed in connection with stringent lockdown measures, their relaxations over time, study designs, etc. A dashboard called SeroTracker has been developed in order to easily visualise global SARS-CoV-2 seroprevalence estimates [26]. They report more than 2,900 seroprevalence estimates from 137 countries, indicating that our study is one of the few that reports several rounds of seroprevalence estimates at a national level for the general population.

## Conclusion

Serial seroprevalence monitoring indicates that in Belgium, a densely populated country in the western Europe, SARS-CoV-2 was introduced throughout the country from the start and the proportion of the infected seropositive population at least doubled within 3 weeks, from 1.8% to 5.3% during the start of the lockdown in spring 2020. In line with reported confirmed cases and COVID-19 deaths, the estimated seroprevalence did not increase further and seroincidence decreased thereafter. The observed decline of the proportion of seropositive people by the end of June would align with reports of quick antibody waning after mild or asymptomatic infection but could also be an issue of change in care seeking behaviour. Serial seroprevalence and seroincidence monitoring in combination with COVID-19 diagnostic testing data can provide a useful tool to estimate the proportion of the population recently infected with SARS-CoV-2. These findings have helped calibrate the Belgian response to the epidemic’s first wave and may guide policymakers to control for potential future waves.

## Data sharing

Data are available on Zenodo (http://doi.org/10.5281/zenodo.4664403).

## Supplementary Data

7565000 Supplement
